# Breast, ovarian, and endometrial malignancies in systemic lupus erythematosus: a meta-analysis

**DOI:** 10.1038/bjc.2011.115

**Published:** 2011-04-12

**Authors:** S Bernatsky, R Ramsey-Goldman, W D Foulkes, C Gordon, A E Clarke

**Affiliations:** 1 Division of Rheumatology, McGill University Health Centre, Montreal, QC, Canada; 2 Division of Clinical Epidemiology, Research Institute of the McGill University Health Centre, 687 Pine Avenue West, V-Building, V2.09, Montreal, QC H3A 1A1, Canada; 3 Division of Rheumatology, Northwestern University, Chicago, IL, USA; 4 Program in Cancer Genetics, Departments of Oncology and Human Genetics, McGill University, Montreal, QC, Canada; 5 Department of Rheumatology, Sandwell and West Birmingham Hospitals NHS Trust, City Hospital, Birmingham, UK; 6 Division of Clinical Immunology/Allergy, McGill University Health Centre, Montreal, QC, Canada

**Keywords:** systemic lupus, SLE, malignancy

## Abstract

**Background:**

An increased lymphoma risk is well documented in systemic lupus (SLE). Less attention has been focused on women's cancers, even though SLE affects mostly females. Our objective was to estimate the risk of breast, ovarian, and endometrial cancers in SLE, relative to the general population.

**Methods:**

Data were included from five recent studies of large SLE cohorts. The number of cancers observed was determined for each cancer type. The expected number of malignancies was ascertained from general population data. The parameter of interest was the standardised incidence ratio (SIR), the ratio of observed to expected malignancies.

**Results:**

The five studies included 47 325 SLE patients (42 171 females) observed for 282 553 patient years. There were 376 breast cancers, 66 endometrial cancers, and 44 ovarian cancers. The total number of cancers observed was less than that expected, with SIRs of 0.76 (95% CI: 0.69, 0.85) for breast cancer, 0.71 (95% CI: 0.55, 0.91) for endometrial cancer, and 0.66 (95% CI: 0.49, 0.90) for ovarian cancer.

**Conclusions:**

Data strongly support a decreased risk of breast, ovarian, and endometrial cancers in SLE. This may be due to inherent differences in women in SLE (*vs* the general population) regarding endogenous oestrogen, other medications, and/or genetic make-up.

An increased risk of lymphoma in systemic lupus erythematosus (SLE) has been the focus of much attention, particularly in the last decade (Gayed *et al*, 2009). However, as SLE is a disease predominantly of women, some of the most prevalent types of malignancies in SLE are actually those that commonly occur in women in the general population – breast, ovarian, and endometrial cancers. Despite this, much less attention has been focussed on these malignancies. Our objective was to estimate the risk of breast, ovarian, and endometrial cancers in women with SLE, relative to an age, calendar year, and geographically matched general population.

## Methods

A literature review was conducted to identify published peer-reviewed articles documenting cancer occurrence in SLE, with a focus on observational cohort designs. It has been established (Furlan *et al*, 2006) that standard systematic literature searches do not effectively identify key manuscripts when observational designs are of primary interest. Thus, as per Furlan *et al* (2006), we completed a progressive literature search. This methodology involves identifying key articles from existing reviews of the literature, and then using search terms (in Medline, MeSH terms) from these citations to locate additional articles. Progressive search strategies locate essentially 100% of articles as compared with traditional systematic literature searches (Furlan *et al*, 2006).

For our analyses, we reviewed all cohort studies published since 1995, which provided basic descriptive information (regarding cohort assembly, demographics, person-time of observation, etc.), and cancer outcomes. The date of 1995 was chosen because the vast majority of the literature regarding cancer risk in SLE (including those with most rigorous methods) has been published in the last 10–15 years. However, several studies of cancer in SLE published since 1995 have come from centres that later contributed data to the international multi-centre cohort study of cancer in SLE, published in 2005 (Bernatsky *et al*, 2005b). Such studies were themselves excluded from our current analyses, as they were essential subset analyses of the larger multi-centre sample. To ensure the quality of the data included, we additionally required that outcome ascertainment was made through cancer registry linkage. The criteria for inclusion in our current analyses were thus as follows: studies of cancer occurrence in SLE cohorts published since 1995, where cancer registry linkage had been conducted to ascertain cancer occurrence, provided they were not replicated in other larger published data sets.

In our primary analysis, we did not consider random effects; the total number of cancers observed across all studies was summed for each of the three different cancer types (breast, endometrial, and ovarian). The total expected number of malignancies for each cancer type, derived in each study from applying general population cancer incidence data to the observed number of patient years of follow-up for each study, was similarly determined. Patients who contributed a cancer of one type could still contribute person-time and events for other cancer types. Our parameter of interest was the standardised incidence ratio (SIR), the ratio of observed to expected malignancies. The 95% confidence interval (CI) for each SIR was estimated by considering the observed number of cancers as a Poisson-distributed variable and finding its related interval from published tables (Breslow and Day, 1987).

The studies examined all patients in the cohort and did not exclude patients with a previous history of cancer. Of course cancer events that had occurred before SLE diagnosis (and cohort entry) were not included, but the patients with a history of previous cancer were still followed and contributed patient-time just like the others, and if they developed a cancer during the follow-up that event was included.

As SIRs for cancer may differ across centres, we also fit a random-effects hierarchical model allowing differences among studies, rather than assuming a single fixed rate across all. Standardised incidence ratio estimation using this hierarchical modelling represents a compromise between the summing of data across sites (our primary analysis, which assumes no variation in cancer experience from one centre to the next) *vs* independent estimates for each centre (the other extreme, which would preclude estimation of a single SIR). We used the Gibbs sampler as implemented with WinBUGS 1.4 software to estimate the model parameters, with 95% credible intervals (CrI) (Gelman *et al*, 1995).

## Results

Of the cohort studies of cancer occurrence in SLE that have been published since 1995, six provided data for the international cohort study published in 2005 (Bernatsky *et al*, 2005b). These smaller subset analyses (Sweeney *et al*, 1995; Abu-Shakra *et al*, 1996; Ramsey-Goldman *et al*, 1998; Sultan *et al*, 2000; Cibere *et al*, 2001; Nived *et al*, 2001) were excluded, whereas the larger international cohort study was included, along with four other large cohort studies from Sweden (Bjornadal *et al*, 2002), Denmark (Mellemkjaer *et al*, 1997), the United States (Parikh-Patel *et al*, 2008), and South Korea (Kang *et al*, 2010).

The five studies identified (Table 1) together included 47 325 SLE patients (42 171 of these female) observed for a total of 282 553 patient years (mean 5.9 years). The overall cancer SIR for each study was in keeping with a slight increase in total cancer risk (which in each study had been demonstrated to be largely driven by an increased risk of haematological malignancy). In total, there were 376 breast cancers, 66 endometrial cancers, and 44 ovarian cancers. In each case, the total number of cancers expected far exceeded that observed, with SIRs of 0.76 (95% CI: 0.69, 0.85) for breast cancer, 0.71 (95% CI: 0.55, 0.91) for endometrial cancer, and 0.66 (95% CI: 0.49, 0.90) for ovarian cancer. Our random-effects model produced very similar findings, with an SIR of 0.76 (95% CrI: 0.68, 0.84) for breast cancer, 0.72 (95% CrI: 0.56, 0.91) for endometrial cancer, and 0.69 (95% CrI: 0.50, 0.90) for ovarian cancer.

## Discussion

In western countries, breast cancer has the highest incidence rate of all non-skin cancers (Curado *et al*, 2007). Endometrial cancer is the most common cancer of the female reproductive system and is one of the top five highest-incident cancers among women in the developed world (Curado *et al*, 2007). Like breast cancer, the risk of endometrial cancer increases with oestrogen exposure, both endogenously and via hormone replacement therapy (Pike *et al*, 1997). In addition, ovarian cancers may be precipitated by oestrogen exposure (Xu *et al*, 2004; Beral *et al*, 2007). In the general population, late menopause can increase both breast and endometrial cancer risk (La Vecchia *et al*, 1984; Peeters *et al*, 1994). Women with SLE have been shown to have earlier menopause (even when not exposed to cytotoxic medications), as compared with women without SLE (Cooper *et al*, 2002). This reproductive factor could be one of the driving forces behind the observed decreased risk of breast, endometrial, and possibly ovarian cancers in SLE. We are currently exploring this in a very large multi-centre study of cancer incidence in SLE (Bernatsky *et al*, 2010). However, because our preliminary data suggest that breast cancer in SLE is decreased in both pre-menopausal and post-menopausal women, we suspect that there are additional factors contributing to a protective effect for certain cancers in SLE.

Other medication exposures are of interest. Hydroxychloroquine is an anti-malarial drug often used in SLE, and this has theoretically some potential to inhibit the growth of breast cancer cells (Rahim and Strobl, 2009). However, one earlier report of a potential beneficial effect of antimalarial agents against malignancy in rheumatic diseases like SLE (Ruiz-Irastorza *et al*, 2007) has not necessarily been supported by other studies (King and Costenbader, 2007; Bernatsky *et al*, 2008a). Intriguingly, although concerns have long existed regarding the carcinogenic potential of immunosuppressive drugs, results from a recent case–cohort study did not establish these agents as independently associated with cancer risk for non-haematological malignancies in SLE (Bernatsky *et al*, 2008b). In fact, there was a trend, although non-significant, towards fewer non-haematological malignancies treated with immunosuppressive agents (the adjusted hazard ratio for overall cancer risk after any immunosuppressive drug was 0.82, 95% CI 0.50–1.36). Again, we are completing additional case–cohort analyses in the context of our expanded multi-centre international cohort study (Ruiz-Irastorza *et al*, 2007), which may be able to identify more precisely the role of these agents (relative to the risk related to the disease itself) in cancer risk for women with SLE.

Genetic factors might also underpin this association breast and ovarian cancers are components of several autosomal dominant cancer syndromes. The syndromes most strongly associated with both cancers are the *BRCA1* or *BRCA2* mutation syndromes (Narod *et al*, 1995), but these do not account for most breast cancers that arise in the general population. Genome-wide association studies have identified common variants (single nucleotide polymorphisms, (SNPs)) at various loci that are associated with an increased risk of breast cancer (Easton *et al*, 2007). Various genetic variants within 8q24 have been linked to breast, prostate, and other cancers (Ghoussaini *et al*, 2008). We are pursuing studies of data from genome-wide association studies in unselected SLE patients, to help to determine whether some of these specific cancer-related SNPs have a lower frequency than expected in persons with SLE.

Regarding overall cancer risk, the data present do not include wash-out periods, but in our large multi-centre cohort we did in sensitivity analyses exclude the first year of SLE diagnosis to account for the potential of paraneoplastic syndromes. This did not change the results. We note that the study cohorts of Mellemkjer, Bjornadal, and Patel *et al* represented SLE patients drawn from hospitalisation records (where the SLE diagnosis was not necessarily clinically confirmed), whereas the study cohorts of Bernatsky and by Kang were drawn from clinical registries (and all subjects had clinically confirmed SLE. However, the follow-up time was not systematically different, and the SIR results were remarkably similar. One discernable difference in terms of demographics between the hospital-based cohorts and the clinical cohorts was that there was a tendency for a slightly greater proportion of males with the hospital-based cohort, but again this did not seem to influence the results.

Considering Table 2, the results are relatively homogeneous, but in some cases the data from Kang *et al* are a bit discrepant. In most cohorts the patients were primarily Caucasian, whereas the study of Kang *et al* featured Asian patients. This suggests that perhaps the cancer profile of SLE patients may be influenced by race. A potential limitation is that we did not have information about cancer risk stratified by race. However, race-specific SIRs for cancers overall have been published using data from the international multi-centre SLE cohort (Bernatsky *et al*, 2005a). Those analyses demonstrated similar SIRs for cancer risk overall in black and white patients with SLE, although precise estimates for Asians could not be demonstrated. As the multi-centre study is being updated, with twice as many patient years as previously, future data may help to resolve the issue (Bernatsky *et al*, 2010).

In summary, the published data to date strongly support a decreased risk of breast, ovarian, and endometrial malignancies in women with SLE. This may be due to inherent differences in women in SLE (*vs* the general population) with respect to endogenous oestrogen or other medications, and/or genetic make-up. Further studies may elucidate this.

## Figures and Tables

**Table 1:** Characteristics of the five recent large cohort studies of cancer in systemic lupus erythematosus

**Study**	**Population**	***N* total**	**Mean person-years follow-up**	***N* females**	**Over-all SIR (95% CI)**	**NHL SIR (95% CI)**
Bernatsky *et al* (2005a, 2005b)	Multi centre clinical	9547	6.0	8607	1.15 (1.05–1.27)	3.64 (2.63–4.93)
Bjornadal *et al* (2002)	Sweden administrative	5715	8.8	4201	1.25 (1.14–1.37)	2.86 (1.96–4.04)
Kang *et al* (2010)	South Korea clinical	914	6.3	914	1.45 (0.74–2.16)	15.4 (2.90–37.7)
Mellemkjaer *et al* (1997)	Danish administrative	1585	6.8	1316	1.30 (1.06–1.58)	5.2 (2.2–10.3)
Parikh-Patel *et al* (2008)	USA administrative	30 478	5.2	27 133	1.14 (1.07–1.20)	2.74 (2.22–3.34)
Total		47 325	5.9	42 171		

Abbreviations: SIR=standardised incidence ratio; CI=confidence interval.

**Table 2:** Breast, ovarian, and endometrial cancers in systemic lupus erythematosus

	**O**	**E**	**SIR**	**95% CI**	
*Breast*					
Bernatsky	73	96.1	0.76	0.60	0.96
Bjornadal	52	72.2	0.72	0.54	0.94
Kang	0	1.2	0	0.00	3.06
Mellemkjer	14	14	1.00	0.55	1.68
Patel	237	311.9	0.76	0.67	0.86
Total	376	496.9	0.76	0.69	0.85
					
*Endometrial*					
Bernatsky	6	16.9	0.36	0.13	0.77
Bjornadal^a^	26	24.4	1.07	0.70	1.56
Kang	1	0.25	3.94	0.00	15.44
Mellemkjer	4	3.1	1.29	0.35	3.30
Patel	29	48.1	0.60	0.40	0.87
Total	66	92.8	0.71	0.55	0.91
					
*Ovarian*					
Bernatsky	9	14.5	0.62	0.28	1.18
Bjornadal	7	14.6	0.48	0.19	0.99
Kang	1	0.38	2.62	0.04	14.65
Mellemkjer	0	3.0	0.00	0.00	1.22
Patel	27	32.8	0.82	0.54	1.20
Total	44	65.3	0.66	0.49	0.90

Abbreviations: SIR=standardized incidence ratio; O=observed cancers; E=expected cancers; CI=confidence interval.

In most cohorts the patients were primarily Caucasian, whereas the study of Kang *et al* featured Asian patients.

a International Classification of Diseases-9 code 171+174.
